# A perspective of randomness in a clinical test of olfactory performance

**DOI:** 10.1038/s41598-023-45135-x

**Published:** 2023-10-20

**Authors:** Jörn Lötsch, Thomas Hummel, Alfred Ultsch

**Affiliations:** 1 Institute of Clinical Pharmacology, Goethe-University, Theodor Stern Kai 7, 60590 Frankfurt am Main, Germany; 2 Fraunhofer Institute for Translational Medicine and Pharmacology ITMP, Theodor-Stern-Kai 7, 60596 Frankfurt am Main, Germany; 3 Smell & Taste Clinic, Department of Otorhinolaryngology, Universitätsklinik Gustav Carl Carus, Technische Universität Dresden, Fetscherstrasse 74, 01307 Dresden, Germany; 4 DataBionics Research Group, University of Marburg, Hans-Meerwein-Straße 22, 35032 Marburg, Germany

**Keywords:** Olfactory system, Statistical methods, Data acquisition

## Abstract

Random walks describe stochastic processes characterized by a sequence of unpredictable changes in a random variable with no correlation to past changes. This report describes the random walk component of a clinical sensory test of olfactory performance. The precise definition of this stochastic process allows the establishment of precise diagnostic cut-offs for the identification of olfactory loss. Within the Sniffin`Sticks olfactory test battery, odor discrimination (D) and odor identification (I) are assessed by four- and three-alternative forced-choice designs, respectively. Meanwhile, the odor threshold (T) test integrates a three-alternative forced-choice paradigm within a staircase paradigm with seven turning points. We explored this paradigm through computer simulations and provided a formal description. The odor threshold assessment test consists of two sequential components, the first of which sets the starting point for the second. Both parts can be characterized as biased random walks with significantly different probabilities of moving to higher (11%) or lower (89%) values. The initial odor concentration step for the first phase of the test and the length of the subsequent random walk in the second phase significantly affect the probability of randomly achieving high test scores. Changing the odor concentration from where the starting point determination for the second test part begins has raised the current cut-off for anosmia, represented as T + D + I < 16, from the 87th quantile of random test scores to the 97th quantile. Analogous findings are likely applicable to other sensory tests that use the staircase paradigm characterized as random walk.

## Introduction

Clinical testing of olfactory function is most commonly performed in ENT or neurology departments where olfactory dysfunction is the symptom leading to consultation or an early sign of disease^1^. Severe acute respiratory syndrome coronavirus type 2 (SARS-CoV2) infections have recently increased interest in olfactory testing^2,3^. Many common olfactory tests consist of a single test or battery that assesses combinations of three different components of the sense of smell, namely (1) the perception of odors at low concentrations (odor threshold), (2) the nonverbal discrimination of different odors (odor discrimination), and (3) the ability to name or associate an odor (odor identification). Some other tests add olfactory memory or other important features (for an overview see e.g.^4^), but these will not be discussed further in this report. One of the established olfactory test batteries that assesses all three components mentioned above is the Sniffin’ Sticks test^5,6^. It is clinically well established and has an extensive record of use in the published biomedical literature. A search of the PubMed database at https://pubmed.ncbi.nlm.nih.gov/ on March 16, 2023 for “(Sniffin’ Sticks) OR (Sniff and Sticks) NOT(review[PT])” returned 1023 results. From the first mention in 1996 in^6^, the use of Sniffin` Sticks in publications per year has steadily increased to a recent maximum of 153 publications in 2022.

Like other olfactory tests, the Sniffin’ Sticks test assesses olfactory function by presenting odors in a forced choice paradigm. The subject must either correctly name an odor known to the examiner from a set of alternatives, or discriminate an odor from also presented blanks. In either case, the test score is determined as the sum of the correct responses. The test result is then used to establish three olfactory diagnoses, i.e., lack of olfactory sensitivity, termed “anosmia”, reduced function, termed “hyposmia”, and normal function, termed “normosmia”. The boundaries defining these diagnoses were found by analyzing the distributions of sum scores in large cohorts of increasing size from n = 1036^7^, with updates derived from testing n = 3282^8^ to n = 9139 subjects^9^. The theoretical limit of anosmia is expected to be a defined confidence limit of results when olfactory test responses are only guesses. This is straightforward for the four-alternative and three-alternative forced-choice tasks, where the probability of guessing the correct result is ¼ and 1/3, respectively, and the confidence limits are easily calculated from common probability equations. However, determining the theoretical limit for the odor threshold is not as straightforward because the three-alternative forced-choice task is embedded in a complex staircase design that defies the application of standard probability equations. Because of this difficulty, previous publications have not reported an odor threshold that marks the 90% change in a guessed score; the threshold is reported only from observed scores in individuals diagnosed as anosmic by other means^7^.

To overcome the empirical component of the diagnostic limits for anosmia, the present analyses approach the problem from the perspective of test results generated by a purely random process. A computational approach has been adopted that combines the analysis of the results of software-coded tests with the subsequent development of a probabilistic description of the underlying stochastic process as a one-dimensional random walk.

## Methods

### Description of the clinical olfactory test algorithm

The Sniffin’ Sticks test uses felt-tip pens containing odors dissolved in odorless propylene glycol or just the solvent. The three subtests, designed to measure the three components of olfactory performance (T, D, I), each use 16 individual tests in different algorithms. While odor discrimination (D) and odor identification (I) are tested with four- and three-alternative forced-choice designs respectively, the odor threshold test (T) embeds a three-alternative forced-choice paradigm in a seven-alternative staircase paradigm. Possible ranges of subtest scores are [1, 16], [0, 16] and [0, 16] for odor threshold, discrimination and identification, respectively. The main outcome of the Sniffin’ Sticks test is the TDI sum score. Pathological olfactory function is indicated by a TDI ≤ 30.5, with the empirical cut-off between hyposmia and anosmia at TDI = 16^9^. The latter is due to the finding in the first multicenter evaluation^7^ that patients diagnosed with anosmia never exceeded a certain TDI value, specifically TDI > 16.

The more detailed test algorithm is as follows. Odor identification and odor discrimination are tested using a multiple forced-choice design, i.e., the tested individual has to choose from a set of options. For odor discrimination, three odors are presented, two of which are identical and one of which, the target, is different from the others. The task is to select the odor that smells different (three-alternative forced-choice design). For odor Identification, a single odor is presented along with a list of four odor names from which one must be selected (four-alternative forced choice design). In both tests, the sum of correct answers out of a total of d = 16 trials is the test result.

The odor threshold test is more complex. It is a three-alternative forced-choice design embedded in a staircase paradigm. An odor is presented at 16 concentrations, from a dilution of 4% and further diluted in a geometric series at a ratio of 1:2. In the Sniffin’ Sticks test addressed specifically in this report the odor is phenylethyl alcohol, which has a rose-like smell. For each dilution, a three-alternative forced-choice design is used in which the diluted odor (target) is presented together with two blanks at 3-s intervals. Which one is the target is randomly chosen by the investigator during the test. The test begins with the lowest concentration (T(0) = 16), where T denotes the threshold score step, i.e., the 16 dilutions of phenylethyl alcohol. After an incorrect response, the concentration is increased by 2 threshold levels (T(1) = 14) until the odor is correctly identified twice in a row at the same concentration. This is considered the starting point (T_start_ = T(n)) of the test and is recorded as the first turning point (staircase paradigm for staring point, SPSP). The odor concentration is then reduced in steps of one threshold score value, i.e., to T(n) + 1, until the odor is no longer detected, i.e., until it is not correctly identified twice in a row. This is the 2nd turning point from which the odor concentration increases again. In this way, two correct identifications in a row or one incorrect identification trigger a reversal of the staircase to the next higher or lower concentration step, respectively. If the test reaches the limit of T = 1, the lowest available odor dilution, the failure to correctly detect the odor is noted as a turning point and the next odor concentration presented is again T = 1. Successfully detecting the odor at T = 1 twice in a row triggers another increase in odor dilutions to T = 2, and so on. Similarly, if the test reaches the limit of T = 16, the highest available odor dilution, success in correctly detecting the odor twice in a row is noted as a turning point and the next odor concentration presented is again T = 16. Failure to detect the odor twice in a row at T = 16 results in T = 15 being presented next, and so on. The test ends after seven turning points. In the standard version of the Sniffin` Sticks, the odor threshold is the average of the last four of seven staircase reversals or turning points (Staircase Paradigm for Threshold, SPT).

### Software coding of the clinical olfactory test algorithm

To study the behavior of the olfactory tests, in particular the consequences of successive random decisions in the odor threshold test that underlie the up and down movements to higher or lower odorant dilution steps during the staircase part of this test, a computer simulation was chosen. Coding was done by one author (JL) in the Python language^10^ using Python version 3.8.15, available free of charge at https://www.python.org (accessed 19 January 2023). The main packages used for the simulations were the numerical Python package “numpy” (https://numpy.org^11^), “pandas” (https://pandas.pydata.org^12,13^). To obtain as unbiased results as possible, the subsequent theoretical description of the olfactory test was independently coded by another author (AU) and in the matrix laboratory language using MATLAB (version 9.13.0.2049777 (R2022b) for Windows™, MathWorks, Natick, MS, USA).

Programming the odor discrimination and odor Identification tests was straightforward. The code for the odor discrimination test is shown as an example in Textbox 1 in the Supplementary Information; the odor identification test was programmed similarly, except that there are four alternatives to choose from instead of three. Programming the odor threshold assessment was more complex. Therefore, two different versions of Python code were written Textbox 2 in the Supplementary Information, based solely on the Sniffin` Stick manual with occasional consultation with the clinical author (TH) for test details. Python variant 1 mimicked the up and down behavior of the staircase test, while Python variant 2 considered the test as a flat sequence of random choices.

To verify that the results of the coded olfactory test were consistent with real observations, a large data set from a previous publication^14^ was used to check whether the simulated data matched the real olfactory data. The dataset contained the results of the Sniffin’ Sticks test from 10,714 subjects who presented with the symptom "loss of smell" at the Clinic for Smell and Taste, Dept. of ENT, TU Dresden, Germany. It originated from a retrospective cross-sectional study that adhered to the Declaration of Helsinki, and was approved by the Ethics Committee of the Medical Faculty of the Technical University of Dresden (number EK251112006). To avoid circular reasoning in the diagnosis of anosmia, the odor threshold was excluded and anosmia was determined from the odor discrimination and identification scores based on the empirical limits given in a publication that published the 90% percentiles of the random results for both tests, along with the advice to use the sum of both limits as the criterion, i.e., D + I < 16^15^.

### Investigation of random results of the clinical olfactory test algorithm

Simulations were run with 100,000 replicates unless stated otherwise. The distributions of the random results of the soft-coded olfactory test were analyzed using transformations along Tukey’s ladder of powers^16,17^. In addition, the significance of specific test algorithm details to random results were analyzed, in particular the starting point and the choice of turning points to be averaged for the final threshold score. Statistical analyses of the results were done in the Python or in the R language^18^. For the latter, the R software package^19^, version 4.2.2 for Linux, which is available from the Comprehensive R Archive Network (CRAN) at https://CRAN.R-project.org/.

## Results

First, the olfactory test was mirrored by a software implementation that produced purely random results on a large scale (e.g., 100,000 simulations), on which the behavior of the test could be studied before the observations were translated into a theoretical probabilistic solution.

### Analyses of results of the soft-coded olfactory test on random choices

#### Correct operation of the software coded olfactory test algorithm for odor threshold assessment

**Figure 1:**
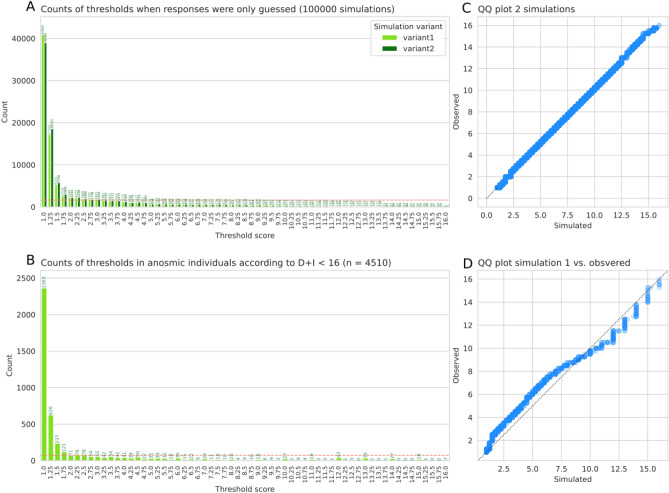
Bar plot of odor thresholds obtained with the software-encoded odor threshold test algorithm implemented in two different Python code variants (**A**) (100,000 simulations) and comparison with observed odor thresholds of 4510 patients (**B**). The red dashed horizontal lines mark the uniform distribution expected if each threshold had been chosen with equal probability. The QQ plots of one simulation variant against the other (**C**) and of variant 1 against the real observations (**D**) show perfect to satisfactory agreement. The figure was created using Python version 3.8.15 for Linux (https://www.python.org), with the seaborn statistical data visualization package (https://seaborn.pydata.org^20^).

**Figure 2:**
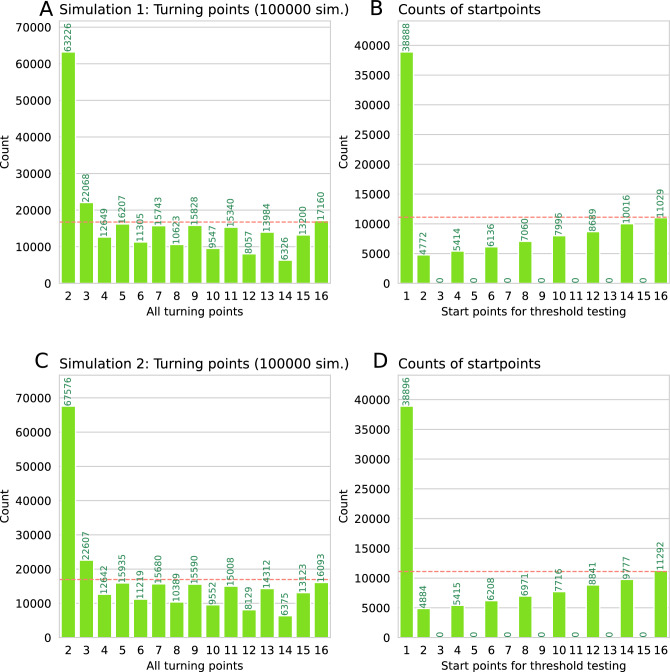
Bar plot of start points (**A** and **C**) and turning points (**B** and **D**) in the with the software-encoded odor threshold test algorithm implemented in two different Python code variants (upper and lower panels, respectively) (100,000 simulations). The red dashed horizontal lines mark the uniform distribution expected if each threshold had occurred with equal probability. The figure was created using Python version 3.8.15 for Linux (https://www.python.org), with the seaborn statistical data visualization package (https://seaborn.pydata.org^20^).

The two Python implementations of the software-encoded test algorithm for the determination of odor thresholds produced practically identical results, as indicated by a quasi-linear QQ plot along the line of identity (Fig. 1). The agreement was also found in the details of the starting points of the olfactory tests and the turning points reached during the run through the test algorithm (Fig. 2). The simulations also agreed with observations of odor thresholds in 4510 subjects who were classified as having an olfactory diagnosis of anosmia based on their odor discrimination and identification scores of D + I < 16.

#### Distributions of soft-coded olfactory tests on random choices

**Figure 3:**
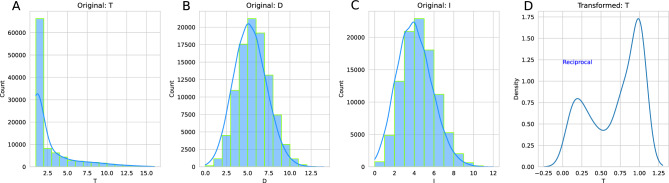
Distribution of results from 100,000 simulations of odor thresholds (**A**), odor discrimination (**B**) and odor identification (**C**). In addition, panel (**D**) shows the probability density of the transformed odor thresholds according to the results of the tests along Tukey's ladder of powers. The figure was created using Python version 3.8.15 for Linux (https://www.python.org), with the seaborn statistical data visualization package (https://seaborn.pydata.org^20^).

Distributions of odor threshold, discrimination and identification were analyzed with 10^5^ simulations. The resulting Box-Cox transformation with values of λ ≈ 1 indicated no transformation for the simulated scores of odor discrimination and identification. In contrast, a Box-Cox λ = − 1.14 for the transformation of the odor thresholds suggested a reciprocal transformation according to the closest points on Tukey’s ladder of powers. Interestingly, when the reciprocal transformation of odor thresholds was performed, the distribution was clearly not unimodal (Fig. 3). A mode at 1/T = 1 was accompanied by a second mode at lower values of 1/T ≈ 1/5. The rejection of unimodality was supported by a highly significant dip test^21^ (dip = 0.086275, p < 10^–293^) using the Python “diptest” package available at https://pypi.org/project/diptest/.

#### Olfactory test score limits for the diagnosis of anosmia based on random choices

**Figure 4:**
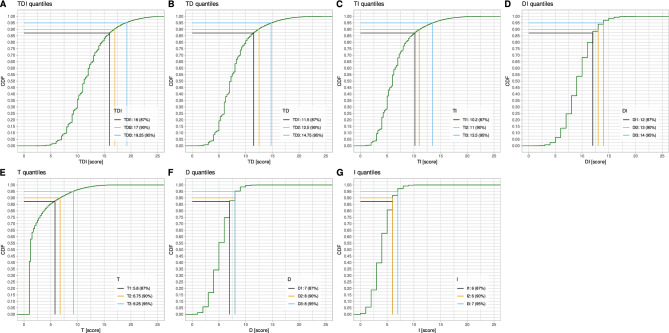
Quantiles of random results from the software-encoded olfactory test in the original test version with odor threshold test start point at T_start_ = 8 instead of T_start_ = 16. The black vertical and horizontal lines indicate the 87th quantile, which is in the original version the accepted limit for anosmia according to the empirical limits given in the actual olfactory test instructions (100,000 simulations). The yellow and blue lines indicate the 90th and 95th quantiles, respectively, for the random results of the soft-coded olfactory test. All combinations of the three subtests, odor threshold, discrimination and identification, T, D and I, respectively, are shown in panels (**A**–**G**). The figure has been created using the R software package (version 4.2.2 for Linux; https://CRAN.R-project.org/ (R Development Core Team^19^)) and the library “ggplot2” (https://cran.r-project.org/package=ggplot2 (Wickham^22^)).

Current recommendations for the Sniffin’ Sticks test use the empirically established criterion of a TDI ≤ 16 as an olfactory diagnosis of anosmia, i.e., a non-functioning sense of smell. The percentiles obtained in 100,000 random results indicate that this cutoff corresponds to the 87th percentile of TDI scores obtained as the sum of randomly generated test results for odor threshold, odor discrimination, and odor identification. In the original test algorithm, the 90th percentile of simulated TDI scores corresponds to TDI = 17 (Fig. 4).

#### Significance of specific details of the odor threshold test to random olfactory test results

##### Starting point of the odor threshold test

**Figure 5:**
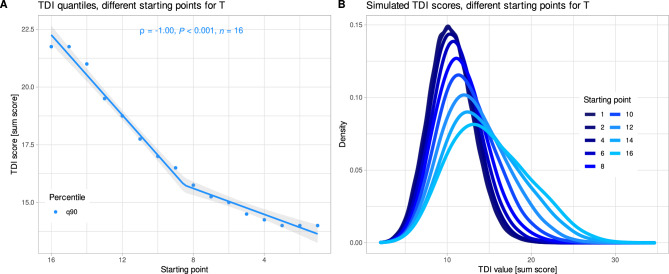
Dependency of the final TDI test score when all responses were guessed from the starting point of the odor threshold test (25,000 simulations). (**A**) Correlation of the 90th quantiles of the TDI with the starting point used to determine the odor threshold. The lines indicate linear splines with breakpoints determined from goodness-of-fit analysis using analysis of variance of the fitted models. (**B**) Probability density plotted using a kernel smoothing function with default settings in the ‘geom_smooth’ method of the R library “ggplot2” (https://cran.r-project.org/package=ggplot2^22^). The lines are colored in darker or lighter blue with increasing starting value of the odor threshold test. The figure was created using the software package R (version 4.2.2 for Linux; https://CRAN.R-project.org/^19^).

The starting point according to the actual test protocol is determined from T = 16 in steps of T = 2, i.e., 16, 14, …, 2, 1. The final TDI score was correlated with the starting point of the odor threshold testing at a value of Spearman’s ρ = − 1 (Fig. 5). The higher the starting point, the more the distribution of final TDI scores was flattened and shifted to the right. That means that the test design involves a chance that a value close to TDI = 16, i.e., the cut-off for anosmia, can be obtained even without any ability to smell just because the test started at T = 16. This called for readjusting the start point, leading to a proposal for a modified test design presented at the end of the “Results” section.

##### Choice of the number of turning points to be averaged for the odor threshold score

**Figure 6:**
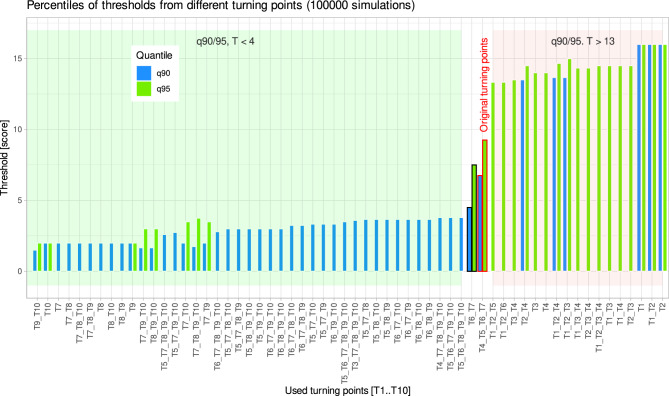
Bar plot of the 90th and 95th quantiles of odor thresholds obtained in a random test scenario by averaging 1–10 turning points (100,000 simulations). The split view shows the turning points associated with the lowest (green shaded area, arbitrarily restricted to T < 4) and highest (red common area, arbitrarily restricted to T > 13) quantile values. Also shown are the 4th—7th turning points used for threshold calculation according to the olfactory test instructions (red framed bares), and in addition if in that standard scenario, only the last two turning points were averaged (black framed bars) The figure was created using the software package R (version 4.2.2 for Linux; https://CRAN.R-project.org/^19^) and the R library “ggplot2” (https://cran.r-project.org/package=ggplot2^22^).

**Figure 7:**
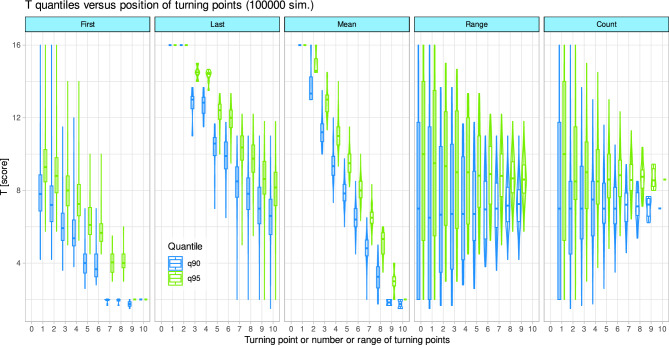
Tunning points when the responses in the olfactory threshold test are guessed. Box plots of the 90th and 95th quantiles of randomly generated odor thresholds, depending on the position of the turning points in a sequence of [1,…,10] turning points, or on the number of turning points used for averaging to the final score. The first and last turning points indicate the beginning and end of the position range of the turning points used. The mean value refers to the average position in the sequence of turning points. The boxes are constructed from the minimum, quartiles, median (solid line within the box) and maximum values. The whiskers add 1.5 times the inter-quartile range (IQR) to the 75th percentile or subtract 1.5 times the IQR from the 25th percentile. The figure was created using the software package R (version 4.2.2 for Linux; https://CRAN.R-project.org/^19^) and the R library “ggplot2” (https://cran.r-project.org/package=ggplot2^22^).

In an experiment with 10 turning points, there are 2^10^–1 = 1023 possible combinations of turning points from which the final odor threshold value can be averaged. The 1024th permutation would be zero turning points. An overview of the quantiles of odor thresholds associated with the use of different turning points for averaging to the final score (Fig. 6) showed that later turning points produced lower random scores, whereas the use of early turning points carried the risk of a high random T score. The current procedure, according to the test instructions, to use the 4th—7th turning point is in the middle of the extremes and would be slightly improved if only the last two turning points were used for averaging instead of the last four. Details of the relationship between the choice of turning point and the thresholds when the test was answered randomly showed that the higher the position of the turning points used, either defined as the first, last or average position of the turning points, the lower the 90th and 95th quantiles of the randomly generated thresholds were (Fig. 7). The position range of thresholds used, or their total number had less influence.

### Translation of the observed test behavior into a theoretical probabilistic solution

In the theoretical description of the olfactory test, the straight-forward tests of odor discrimination and odor identification were described using standard probabilistic equations, while the focus was on describing the odor threshold test. This required capturing the two parts of the test, determining the starting point for the subsequent part of the test, and then obtaining the subsequent turning points. The result should be olfactory scores consistent with the simulations above, including the importance of the starting point for the second part of the test procedure, the choice of turning points for averaging to the threshold score, and the explanation of the bimodal distribution of TDI scores generated when the olfactory test was performed with random choices.

#### Description of the odor identification and discrimination tests as probability function of the forced choice test design

Here, a standard equation applies. The probabilities of the random results, when the tests of odor discrimination or of identification are completely guessed, are given as a binomial probability distribution with $P = \frac{n!}{{k!*\left( {n - k} \right)!}}*p^{k} *\left( {1 - p} \right)^{{n {-} k}}$ where n is the number of trials of n = 16 odors/pairs, k is the number of correct test responses, and p is the probability of a correctly guessed response, with $p = 0.\overline{3}$ in the odor discrimination test and p = 0.25 in the odor identification test, according to the three- or four-alternative forced-choice designs of the discrimination and identification tests, respectively.

#### Description of the odor threshold test as a random walk

**Figure 8:**
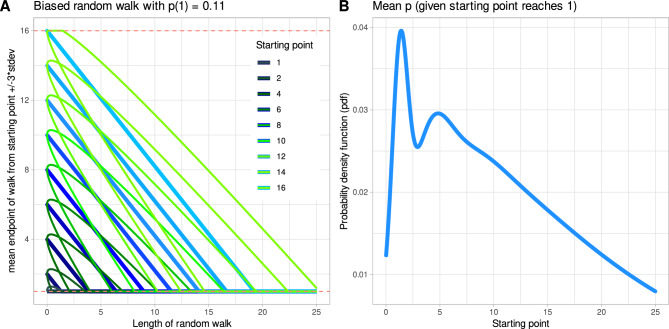
Description of the olfactory test as a random walk: (**A**) Distributions of the evolving mean threshold and the 99.7% limit of expectations. The red dashed lines indicate the boundaries of the random walt at T = 1 and T = 16. (**B**) Expected distribution of odor thresholds integrated over all distributions shown in panel A. The figure has been created using the R software package (version 4.2.2 for Linux; https://CRAN.R-project.org/ (R Development Core Team^19^)) and the library “ggplot2” (https://cran.r-project.org/package=ggplot2 (Wickham^22^)).

The starting point of the odor threshold test, according to the current test protocol, is determined from T = 16 in steps of T = 2, i.e., 16, 14, …, 2, 1. The much less common alternative is a starting point at T = 15 with subsequent steps of T = − 2, which was not considered further due to its infrequent use. The odor concentration step correctly detected on the first two consecutive times is the starting point T_start_ of the test. If this is not the case after eight trials, the subject has reached a value of T_start_ = 1 on the staircase. In fact, guessing with this algorithm usually leads to T_start_ = 1, which was the intended behavior during test development. However, the empirical probability of a higher starting point decreases from 16 to 2 (Fig. 8). Starting points with T_start_ = 16 occur in (1/3)^2^ = 11% of the randomly generated test results. The starting point is also taken as the first turning point of the odor threshold test, according to the test design. Once the starting point is determined, odor thresholds are assessed using a staircase paradigm with steps up and down to higher or lower odorant concentrations. For a purely random outcome of an odor threshold test (random guessing), both processes can be described as random walks as follows.

The determination of the turning points, including the starting point, when the responses to the odor threshold test tasks are only random choices, is given by a random walk with probability p for decreasing the concentration of the next test step and q = 1−p as the probability for increasing the concentration of the next test. Assuming independence of successive olfactory trials, which is reasonable if the subject cannot smell at all, p can be calculated as the probability of randomly guessing 1 out of 3 (= 1/3) on two successive trials, p = (1/3)^2^ = 11.11%. For this type of so-called biased random walk, theoretical results exist and can be used^23–25^.

Let t = 0,…,N (time) be the length of the Biased Random Walk (BRW) i.e., the number of sniff tests with a guessed result. The time t = 0 is the start time of the BRW, which starts at T_start_. For the staircase paradigm to determine the starting points (SPSP), the step width is S_w_ = 2 and the initial starting point is T_start_ = 16 at t = 0. The evolution of mean expectation and the 99.7% range in this algorithm shown in Fig. 8. For the staircase paradigm at threshold T, T_start_ is the result of the start point finding (SPSP) and S_w_ = 1. The distribution of the threshold pdf(t) reached at t can generally be calculated as a Gaussian pdf(t) = N(M,V) = N(M(t),V(t)), with mean M(t) = 2pt− s_w_t + T_start_ and variance V(t) = pqt.

**Figure 9:**
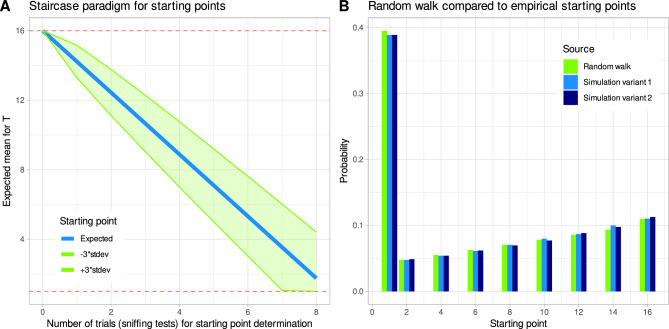
Staircase paradigm for finding the starting point of the odor threshold test: (**A**) Mean expectation of starting points and 99.7% range. (**B**) Comparisons of starting points computed using the random walk implementation of the test with starting points observed in 100,000 simulations using the two Python code variants of the software implementation of the odor threshold test (compare Fig. 3. The figure has been created using the R software package (version 4.2.2 for Linux; https://CRAN.R-project.org/ (R Development Core Team^19^)) and the library “ggplot2” (https://cran.r-project.org/package=ggplot2 (Wickham^22^)).

The integral over all starting point searches for 100,000 trials yields the distribution shown in Fig. 3B and D, respectively for the staircase paradigm for starting points in Fig. 9. With this distribution of T_start_, random walk theory yields the distributions of the evolving mean threshold and the 97% limit and as an integral over all distributions, both shown in Fig. 8. This confirms the existence of a second mode (region of high density) at T ≈ 5, which was found empirically (Fig. 3D). The MATLAB implementation of the theoretical description of the odor threshold test is shown in (Textbox 3 in the Supplementary Information).

#### Comparison of theoretical expectations and empirical observations using the odor threshold test

Finally, to verify whether the theoretical model correctly describes the olfactory threshold test, the previously independent analyses, i.e., the empirical analyses of the soft-coded odor threshold test and the coding of the theoretical description of the underlying stochastic process, were compared. This first confirmed that odor thresholds generated by a pure random process in the olfactory test are bimodally distributed (Fig. 9). The second mode appears approximately at T = 5, which corresponds to the empirical observation in the software-encoded odor threshold test (compare Fig. 3). Second, the starting points with a maximum frequency at T = 1, but also with a decreasing frequency from T = 16 to T = 2 followed exactly the expectation from the description of the starting point determination as a random walk (Fig. 9). Therefore, it can be assumed that the theoretical description of the odor test, particularly the odor threshold subtest, correctly captures the true test.

### Proposed test modification to reduce the likelihood of high random scores

Simulations and their theoretical translation have pointed to a crucial part of the test where improvements can be made to make it less likely that an anosmic person will pass the test with the result of being able to smell, although this was generated only by a rare series of correct guesses. That is, the strongest association of accidentally high TDI values is with the initial starting points of the threshold tests. The design of starting with the highest dilution of T_start_ = 16 was chosen in the original test to avoid premature adaptation and habituation of the subject, which was feared when starting with the highest concentration. However, based on the current assessments, it appears that a few individuals may be misclassified as “non-anosmic” in retrospect, despite their complete inability to perceive odors. The established cut-off for anosmia, set at a score of TDI = 16, corresponds to the 87th percentile of random results, marginally deviating from the originally intended 90th percentile that was based on empirical assessments in clinically likely anosmic individuals^15^.

Having the theoretical basis, the 90th percentile can be obtained at TDI = 17, allowing retrospective adjustments. To improve the test and minimize the likelihood of erroneous rejections of the anosmia diagnosis due to guessed test responses, two different alternatives are available. First, adjusting the anosmia cut-off to TDI = 17 is a viable option. Second, maintaining the existing cut-off at TDI = 16 used in all Sniffin’ Sticks test battery publications so far, but refining the test procedure to ensure that anosmia diagnoses do not fall below the 90th percentile of random results in the test score is another viable approach. For the latter option, an immediately obvious conclusion from the observed test behavior would have been to start the test at T_start_ = 1 in the future. This would minimize the likelihood of obtaining a high test score by chance, consistent with the findings presented above. However, T_start_ = 1 was not chosen for two main reasons. First, starting the test at the highest odorant concentration would expose individuals with a normal sense of smell to frequent exposure to strong olfactory stimuli. This increased exposure could lead to adaptation and habituation processes that could potentially bias the test results. Second, the goal was to keep the test application as short as possible. Starting at T_start_ = 1 would mean that a significant portion of the initial test phase would be wasted, as individuals with a normal sense of smell would easily discriminate the true odorant from the blanks until the dilution approached their actual olfactory threshold. The choice of a new starting point therefore required a compromise between minimizing adaptation/habituation effects, reducing overall test duration, and avoiding the unintended consequence of a higher starting point artificially increasing the chances of achieving a better test score regardless of an individual’s actual sense of smell.

Linear splines were used to examine the relationship between the Threshold Discrimination Identification (TDI) values, which mark the 90th percentile of the final test score obtained by guessing, and the starting points. This analysis revealed a two segmented association, supported by significant goodness-of-fit tests for 1–4 linear spline segments (Fig. 5A). A notable inflection point in the slopes of the 90th percentile regression lines, which defines the normal test values according to the original test design, was observed at approximately T = 8. From this point on, the dependence of the final TDI values on the starting point of the threshold test decreases (Fig. 5A). This particular point, which is centrally located within the range of starting points, also proved to be an intuitive choice. It struck a balance between minimizing the likelihood of random high scores (Fig. 5B), reducing adaptation/habituation effects, and keeping the test duration reasonably short.

**Figure 10:**
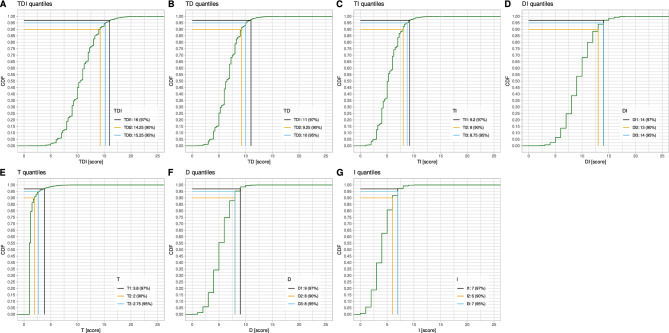
Quantiles of random results from the software-encoded olfactory test in the proposed odor threshold test modification with beginning the finding of the test’s start point at T_start_ = 8 instead of T_start_ = 16. The black vertical and horizontal lines indicate the 96th quantile, which is in this version the accepted limit for anosmia according to the empirical limits given in the actual olfactory test instructions (100,000 simulations). The yellow and blue lines indicate the 90th and 95th quantiles, respectively, for the random results of the soft-coded olfactory test. All combinations of the three subtests, odor threshold, discrimination and identification, T, D and I, respectively, are shown in panels (**A**–**G**). The figure has been created using the R software package (version 4.2.2 for Linux; https://CRAN.R-project.org/ (R Development Core Team^19^)) and the library “ggplot2” (https://cran.r-project.org/package=ggplot2 (Wickham^22^)).

Based on this reasoning, T_start_ = 8 instead of T_start_ = 16 is proposed, with no further changes in the test algorithm from the original version. The start point search is only performed in the direction to T = 1. If T = 8 is detected twice in a row, the following staircase part of the test starts immediately. Corresponding simulations showed that with the proposed modification, n = 4107 (4.1%) instances were above the limit of anosmia at TDI = 16 by accident. TDI = 16 now represents the 97th percentile of random results. Expressed in terms of test performance to detect anosmic subjects, the modification would increase the accuracy from 80.1 to 95%. The availability of a theoretical background and the ability to fully simulate the outcomes of the olfactory test when run with random choices only allows cut-off limits for anosmia to be calculated for each possible combination of the three olfactory subtests. The corresponding values for all combinations of the T, D and I subtests are shown in Fig. 10 for the modified test algorithm.

## Discussion

Random walks are a common phenomenon that occur in many areas, such as molecular motion, the growth of bacterial colonies, the movements of microorganisms^26–29^. One-dimensional random walks are also a standard approach to analyzing stock price movements^30–32^. An early description of random walks goes back more than 100 years^33^, and several modifications and specifications have been added, from which the current description of the olfactory test could be derived. The present clinical test for assessing odor thresholds can be described as one-dimensional biased random walks (BRW) with highly unbalanced probabilities for upward (11%) and downward (89%) movements. The walk is complicated by the nesting of two components, the first consisting of the determination of the starting point for the next walk, which consists of the determination of the subsequent turning points for threshold calculation. The first component is unidirectional, i.e., the movement can only go in the direction of lower values. The second part of the test is an up and down movement.

The formal solution and the empirical soft-coded experiments consistently pointed to the starting point as a critical determinant of the subsequent turning points that ultimately determine the outcome of the odor threshold test. This is consistent with the inclusion of the starting point in an early formalization of random walks^33^ as $X_{n} = x_{0} + \mathop \sum \limits_{j = 1}^{n} Z_{j}$, where $\left( {X_{n} } \right),{{n \in {\mathbb{N}}_{0} }}$ describes the stochastic process leading to the actual position in the walk after j steps from the start point ${x}_{0}$ In the present formalization (see “Results” section), the main determinants of the final results in terms of threshold score obtained by random choices are (1) the starting point, T_start_, (2) the length of the walk, t, and the probabilities in the bias component.

Starting from a high position involves a non-negligible chance of staying in high positions, even if the ability to smell is lacking, i.e., the ability that which would drive an upward movement of the test results indicating that lower odorant concentrations can still be smelled. According to the rules of the test, the probability of a downward movement is still quite high (11%). On the other hand, if the starting position is low, it is very difficult to reach a high position by chance. Thus, according to the actual test instructions, starting at T = 16 includes a non-negligible chance of remaining without olfactory function at higher odor dilutions in the subsequent test, which applies to all published and unpublished applications of the Sniffin’ Sticks test to date. As a result, the actual boundary of anosmia at TDI < 16 marks the 87th percentile. This behavior can be corrected by starting at T = 8, which is a result of the present analyses and raises the cutoff of anosmia at T < 16 to the 97th percentile of randomly generated TDI scores. Finally, the starting point alone, without the subsequent biased random walk, proved to be an inadequate approach to preventing guessed high TDI scores. If only the first part of the random walk, used to determine the starting point, was repeated 10 times and the average of these starting points was used as the threshold, the 90th percentile of the TDI scores would increase to TDI >  = 20.

The formal solution and the empirical soft-coded experiments also emphasized that the length of the random walk, denoted as time t in the formula given in the “Results” section of this report, is an important factor in the actual position in the staircase, which over time has a greater chance of being among the lower scores due to the unbalanced probabilities. In the current experiments, it appears that the use of later turning points for averaging to the final odor threshold shifts the 90th percentile to lower values, i.e., produces the desired reduction in the probability of high test scores achieved by mere guessing at the test. However, a clinical trial that is performed on a patient cannot be continued for an indefinite period of time. In fact, the focus of olfactory test development over the past two decades has been on reducing test burden rather than increasing specificity to detect true loss of olfactory function, triggering proposals of so-called “short” olfactory tests^34–39^. Therefore, shifting the relevant turning points was considered second only to shifting the starting point in the present proposal to reduce the likelihood of false rejections of the diagnosis of anosmia due to chance results. Nevertheless, the present experiments indicate that attempts to shorten the olfactory threshold test by using earlier turning points^39^ should be undertaken with great caution. Given the importance of short test times in clinical practice, the change in probabilities of the bias component of the random walk was not further analyzed. Extending the forced-choice design beyond the current 3-alternative variant would certainly increase testing time and could at best be a rescue measure if other means fail, which, as discussed above, was not the case.

A proposed test adjustment involves shifting the initial starting point for rough threshold determination in the first phase of the testing paradigm from T_start_ = 16 to T_start_ = 8. This change is intended to reduce the likelihood of obtaining falsely high threshold test scores, particularly evident in individuals with complete anosmia, as substantiated throughout this report, while keeping the original published cut-off, as an alternative preferred to moving the cut-off which would imply comprehensive publication of new normative values for the Sniffin’ Sticks test battery. For individuals with a true olfactory acuity greater than T = 8, i.e., normosmic individuals, this adjustment will cause an immediate change in the direction of testing toward values greater than T = 8 from the outset. Subsequently, the second phase of the test, using the up-and-down staircase paradigm, will proceed as in the original version. It is important to note that we can’t simulate the consequences of this adjustment precisely because we don’t have valid estimates of the probability at which normosmic or hyposmic individuals will correctly identify odorant dilutions just at the level of their individual odor thresholds. The present simulations are based on the assumption of completely absent olfactory function and purely random responses in the threshold test. Randomly correct responses in subjects with preserved olfactory function, as experienced in the original test, will still occur, albeit partially mitigated by the repeated testing implemented by the seven staircase reversals implemented in the original test for this very reason. Moreover, with preserved olfactory function, most of the test range will not fall under random responses but the individuals are giving correct responses based on olfactory perception. As such, we anticipate that this adjustment will have minimal impact on the test results for non-anosmic subjects. The authors do not foresee that the test modification will have a large effect on the ratings given by normosmic individuals. However, it is important to note that anosmia often becomes a focal point in medico-legal cases, particularly those involving claims for compensation following accidents that result in loss of olfactory function. In such cases, it is important to minimize the likelihood of erroneously rejecting a diagnosis of anosmia. Nevertheless, a full comparison between the original test design and the modified version may be explored in a future empirical study, which can also address which of the alternative amendments of the test, shifting the anosmia cut-off to TDI = 17 or shifting the start point to T_start_ = 8 to prefer. However, this is beyond the scope of the present evaluation, which focuses primarily on the theoretical framework of staircase odor threshold assessment.

The odor threshold subtest of the Sniffin’ Sticks clinical olfactory test battery is not an isolated design in which random walks have been adapted. Sensory testing with random walks is rather common without the processes being named. In a recent report on changes in point pressure sensitivity as an early sign of Parkinson's disease, the authors specifically described the test design as a “state-of-the-art forced-choice staircase threshold test paradigm”^40^. Similarly, the determination of pain thresholds to mechanical or electrical noxious stimuli in a human experimental study was performed using a forced staircase paradigm similar to the olfactory test analyzed here^41^. Another example is the determination of cuff pain tolerance using a staircase paradigm^42^. The use of staircase paradigms for sensory testing extends to visual or acoustic stimuli for which the detection threshold in chicken has been determined using a staircase paradigm^43^. These are all random walks, although this type of process is barely mentioned by name in the sensory research context. Interestingly, a search for “(“staircase paradigm”) AND (“random walk”)” returned an empty hit list suggesting that the connection between two made in the present report is original.

## Conclusions

In the present analyses, the conceptual basis of the popular staircase paradigm on which several sensory tests, including the present one, are based has been reconceived as a random walk. This makes it possible to assess results obtained by guessing and to set precise limits of anosmia, which were previously based on empirical findings. Regarding randomness in a clinical olfactory performance test, one of its three components, the olfactory threshold test, was found to be a combination of random walks of a special type. To prevent the test from being too easy to pass with a high score, two consecutive trials with a choice of one out of three must be achieved. Statistically, this results in asymmetric probabilities of (1/3)^2^ = 11% to achieve a higher score and 89% to achieve the next lower score. For pure guessing, the odor threshold test results in two successive biased random walk trials. In the present analyses of this process, empirical and formal approaches were applied independently and partly successively, partly in parallel. The concordant results provided mutual support for their correct implementation. This led to the proposal of a modification of the olfactory threshold test, which consists in shifting the starting point to the middle range of scores in order to reduce the risk of erroneously rejecting the diagnosis of anosmia based on purely guessed high scores in the odor threshold test. The availability of a theoretical background of random results in all three subtests of the Sniffin` Sticks test now makes it possible to establish precise cut-off values for the diagnosis of anosmia, i.e., the absence of olfactory function, that capture the test results possible by random selection at defined confidence limits. In addition, recognizing the staircase paradigm used in a variety of sensory and similar tests as a random walk provides a basis for estimating possible unexpected consequences of test modifications.

### Supplementary Information

Supplementary Information 1.

Supplementary Information 2.

## Data Availability

The main parts of the Python and MATLAB code written to perform the present experiments and formal implementations are available as a part of this report (Tables 1—3).
