# Supplementary material for: A perspective of randomness in a clinical test of olfactory performance
Source: Sci Rep. 2023 Oct 20;13:17923. doi: 10.1038/s41598-023-45135-x (PMC10589308; doi:10.1038/s41598-023-45135-x)
Supplement: Supplementary file 1 — Supplementary Information 1. [file 41598_2023_45135_MOESM1_ESM.pdf]

Supplementary Information

Textbox 1: Python code for the simulation of random results in the odor discrimination test (JL). The odor identification test was implemented in a similar way, except for a four-alternative choice paradigm (line 5: “np.zeros((16, 4)”, line 7: l = (1, 0, 0, 0), line 12: randint(4)).

```
1.  # %% Odor discrimination
2.
3.  Discrresult = []
4.  for res in range(nPermut):
5.      correct = np.zeros((16, 3))
6.      for i in range(16):
7.          l = [1, 0, 0]
8.          random.Random(res + i).shuffle(l)
9.          correct[i, :] = l
10.     sumcorrect = 0
11.     for i in range(16):
12.         sumcorrect += correct[i, np.random.RandomState(res + i).randint(3)]
13.     Discrresult.append(sumcorrect)
```

Textbox 2: Python code with two different programming implementations (StaircaseVariant == 1 or 2, starting at lines 5 and 79, respectively) to simulate random results in odor threshold testing (JL).

```
1.  Startfrom = 16
2.  StartSteps = 2
3.  Alternate1516 = False
4.
5.  StaircaseVariant = 1
6.
7.  # %% Odor threshold staircase
8.
9.  # Guessing function
10.
11.
12. def ct1t2():
13.     t1 = [1, 0, 0]
14.     random.Random(res).shuffle(t1)
15.     t1t2 = t1[np.random.randint(3)]
16.     if t1t2 > 0:
17.         t2 = [1, 0, 0]
18.         random.Random(res).shuffle(t2)
19.         t1t2 = t1t2 + t2[np.random.randint(3)]
20.     return(t1t2)
21.
22.
23. Thresresult = []
24. dfThrTurningpointAll = pd.DataFrame(np.zeros((nPermut, 500)))
25.
26. if StaircaseVariant == 1:
27.     # Odor threshold staircase 1
28.
29.     def findStart(t1, StartSteps):
30.         if StartSteps == -99:   # -99 means use given value from Startfrom
31.             Start = t1
32.         else:
33.             ThresStart = []
34.             Start = t1
35.             for i in range(math.ceil(t1 / StartSteps)):
36.                 ThresStart.append(ct1t2())
37.             if max(ThresStart) > 1:
38.                 Start = Start - StartSteps * ThresStart.index(2)
39.             else:
40.                 Start = 1
41.             Start = max(1, Start)
42.         return(Start)
43.
44.     for res in range(nPermut):
45.
46.         if Startfrom == 16 and Alternate1516 == True:
47.             if list(np.random.RandomState(res).choice([0, 1], size=1, p=[0.5, 1-
    0.5]))[0] == 0:
48.                 Startfrom -= 1
49.
50.         Start = findStart(Startfrom, StartSteps)
51.         ThrTurningpoint = [Start]
52.
53.         Thr = Start
54.
55.         for i in range(8):
56.
57.             if Thr < 16:
58.                 Thr += 1
59.             while ct1t2() == 2:
60.                 Thr += 1
61.                 Thr = Thr if Thr < 16 else 16
62.                 if Thr == 16:
63.                     ThrTurningpoint.append(Thr)
64.             ThrTurningpoint.append(Thr)
65.
66.             if Thr > 1:
```

```
                Thr -= 1
            while ct1t2() < 2:
                Thr -= 1
                Thr = Thr if Thr > 1 else 1
                if Thr == 1:
                    ThrTurningpoint.append(Thr)
            ThrTurningpoint.append(Thr)

        Thresresult.append(np.array(ThrTurningpoint[3:7]).mean())
        for i, tur in enumerate(ThrTurningpoint):
            dfThrTurningpointAll.iloc[res, i] = tur

elif StaircaseVariant == 2:
    # Odor threshold staircase 2

    for res in range(nPermut):

        if Startfrom == 16 and Alternate1516 == True:
            if list(np.random.RandomState(res).choice([0, 1], size=1, p=[0.5, 1-0.5]))[0] == 0:
                Startfrom -= 1

        ThresChangeAll = []
        for i in range(400):
            if ct1t2() < 2:
                ThresChangeAll.append(-1)
            else:
                ThresChangeAll.append(1)

        if StartSteps == -99:
            ThresChange = ThresChangeAll
            Start = Startfrom
        else:
            ThresChangeStart1 = ThresChangeAll[:math.ceil(
                    Startfrom / StartSteps)]
                if max(ThresChangeStart1) > 0:
                    ThresChangeStart = ThresChangeStart1[:ThresChangeStart1.index(
                        1)+1]
                    ThresChange = ThresChangeAll[ThresChangeStart1.index(1)+1:]
                    Start = Startfrom - StartSteps * ThresChangeStart.index(1)
                else:
                    Start = 1
                    ThresChange = ThresChangeAll[math.ceil(
                        Startfrom / StartSteps):]

            Thr = [Start, Start + 1 if Start < 16 else Start]
            for i in ThresChange:
                ThrBefore = Thr[-1]
                ThrNew = ThrBefore + i
                if ThrNew < 1:
                    ThrNew = 1
                if ThrNew > 16:
                    ThrNew = 16
                Thr.append(ThrNew)

            ThrDelta = Thr[1:]
            ThrDelta.append(0)

            Thx = []
            for i in range(len(Thr)):
                Thx.append(ThrDelta[i] - Thr[i])

            TurningpointYes = [1]
            for i in range(1, len(Thx)):
                if Thx[i] + Thx[i-1] == 0:
                    TurningpointYes.append(1)
                else:
                    TurningpointYes.append(0)

            Thr1or16 = [i for i, val in enumerate(Thr) if val == 1]
            tur16 = [i for i, val in enumerate(Thr) if val == 16]
            Thr1or16.extend(tur16)

            for i in Thr1or16:
                TurningpointYes[i] = 1
```

```

            ThrTurningpointIdx = [i for i, val in enumerate(
                TurningpointYes) if val == 1]
            ThrTurningpoint = list(np.array(Thr)[ThrTurningpointIdx])

            Thresresult.append(np.array(ThrTurningpoint[3:7]).mean())
            for i, tur in enumerate(ThrTurningpoint):
                dfThrTurningpointAll.iloc[res, i] = tur

    else:
        print("Which variant of staircase implementation?")
```

Textbox 3: MATLAB code for the theoretical description of the odor threshold test as a random walk (AU).

```
clear all; close all; ProgramName ='StaircaseParadigmStartingPoints.m'
Comment = ['Staircase Paradigm for Starting Points(SPSP)']
p =0.33^2;
q = 1-p;
StepWidth = 2;

Tstart = 16;
MaxT   = 8 ;
t = [0:MaxT]';
downt = flipud(t)+1;
NrOfT = length(t);
M      = Tstart+ 2*p*t- t*StepWidth;
ProbT = ((downt/exp(1)+t)*p^2);
ProbT(end) = p;
ProbT(1)    = 1-sum(ProbT(2:end));
Variance = p*q*t;
S =sqrt(Variance);

MplusS = M + 3*S;
MminusS= M - 3*S;
MminusS= max(1,MminusS);
```

```
clear all; close all; ProgramName ='StaircaseParadigmThresold.m'
Comment = ['Staircase Paradigm for Thresolds(SPT)']

% Start probabilities from Staircase Paradigm for Starting Points(SPSP)
StartPoints     = [  1,        2,            4,   6,          8,        10,       12,     14, 16]';
StartPointProb = [0.40635,0.046761,0.054258,0.061754,0.069251,0.076747,0.084244,0.09174,0.11];

p =(1/3)^2;
q = 1-p;
L = 1 ;
R = 16 ;
T = [0:0.01:25]';

NrOfStarts = length(StartPoints);
AllProb = T*0;
 for i=1:NrOfStarts
    StartPkt    = StartPoints(i);
    StartProb = StartPointProb(i);
    MeanN1    = p*T ;
    VarianceN1 = T*p*q;
```

```
        SdevN1     = sqrt(VarianceN1);
        MeanDistN = 2*MeanN1-T;
        MeanN1    = MeanN1    +StartPkt;
        MeanDistN = MeanDistN +StartPkt;
        MplusS = MeanDistN  + 3*SdevN1;
        MminusS = MeanDistN - 3*SdevN1;

        M1ind = min(find( MeanDistN<=L));
        Mleft = T(M1ind);
        Sleft = SdevN1(M1ind)*i %
        pdfL  = normpdf(T,Mleft,Sleft);
        pdfL(isnan(pdfL))=0;
        WeightedPdfL = pdfL*StartPointProb(i);

        AllProb = AllProb+WeightedPdfL;
    end; % for i
```
