# Supplementary material for: A perspective of randomness in a clinical test of olfactory performance
Source: Sci Rep. 2023 Oct 20;13:17923. doi: 10.1038/s41598-023-45135-x (PMC10589308; doi:10.1038/s41598-023-45135-x)
Supplement: Supplementary file 2 — Supplementary Information 2. [file 41598_2023_45135_MOESM2_ESM.docx]

**Supplementary information**

The supplementary information includes Python and MATLAB code from the computational experiments presented.
